# A double mutation of BRAF L597Q and V600E in situ and solitary brain metastasis of occult papillary thyroid carcinoma: A case report

**DOI:** 10.1097/MD.0000000000024458

**Published:** 2021-02-12

**Authors:** Ling Chen, Yue Wu, Huili Bai, Huandong Liu, Xiaosong Li

**Affiliations:** a Doctor of Medicine, Key Laboratory of Molecular Biology of Infectious Diseases, Ministry of Education, Chongqing Medical University; b Bachelor of Medicine, Oncology Department; c Master of Medicine, Clinical Molecular Medicine Testing Center, The First Affiliated Hospital of Chongqing Medical University, Chongqing; d Bachelor of Medicine, Department of Neurosurgery, People's Hospital of Tibet Autonomous Region, Lhasa, China.

**Keywords:** brain metastasis, next generation sequencing, papillary thyroid carcinoma, thyroid cancer

## Abstract

**Rationale:**

The rare BRAF L597Q (c.T1790A) point mutation has been previously reported in childhood acute lymphoblastic leukemia. We present the first rare case of occult papillary thyroid carcinoma with BRAF L597Q mutation in a Tibetan patient.

**Patient concerns:**

A 57-year-old male patient presented with a protruding mass on the left forehead for 2 years and numbness in the right limb for 3 weeks.

**Diagnoses:**

The patient had a double mutation of BRAF L597Q and V600E in 2 separate lesions at thyroid and brain, the immunohistochemical staining showed that the cytokeratin (CK), thyroglobulin (Tg) and thyroid transforming factor-1 (TTF-1) were immunoreactive. All the findings supported the diagnosis of solitary brain metastasis of occult papillary thyroid carcinoma.

**Interventions:**

The patient underwent left frontal lobe metastasis (thyroid cancer) resection that involved craniectomy and artificial skull repair.

**Outcomes:**

During the 24-month follow-up, no postoperative complications or recurrence and metastasis were found.

**Lessons:**

This is the first case of solitary brain metastasis of occult papillary thyroid carcinoma with double mutation of BRAF L597Q and V600E in 2 separate lesions reported in the literature. Our study extends the disease spectrum of occult papillary thyroid carcinoma and suggests that the BRAF L597Q mutation might play a specific role in inducing the solitary brain metastasis of occult papillary thyroid carcinoma in a Chinese Tibetan patient, but the detailed molecular mechanism remains to be confirmed by a large number of functional experiments and clinical research.

## 1 Introduction

The global incidence of thyroid cancer (TC) continues to rise sharply, mainly due to the advent of advanced molecular diagnostic technologies.^[1]^ The 4 major subtypes of thyroid cancer are papillary thyroid carcinoma (PTC), anaplastic thyroid carcinoma, follicular thyroid carcinoma and medullary thyroid carcinoma.^[2]^ PTC is the most common solid tumor in the human endocrine system, which occurs in more than 90% of all thyroid malignancies, and the incidence of PTC has increased rapidly in recent years.^[3–6]^

According to the National Comprehensive Cancer Network (NCCN) Guidelines Insights (Thyroid Carcinoma, Version 2.2018.) and other studies,^[7,8]^ screening for thyroid cancer has been divided into routine testing and molecular testing. The routine testing is accomplished by radionuclide scanning, B-ultrasound, magnetic resonance imaging (MRI), X-ray examination, computed tomography (CT), and thyroid-related hormone detection. The molecular testing consists of genetic testing or testing of other molecular markers by fine-needle aspiration (FNA). The FNA with ultrasound guidance is gradually becoming the preferred method for assessing suspicious thyroid nodules. According to the 2017 Bethesda System for Reporting Thyroid Cytopathology, the cytological examination of FNA specimens is usually divided into 6 categories.^[9,10]^ In recent years, Chinese scholars have established a new sensitive imaging technique, which uses deep convolutional neural network (DCNN) models to increase the diagnostic accuracy by analyzing the sonographic imaging data of clinical ultrasounds.^[11]^

Some gene mutations^[12–15]^ have played an important role in the proliferation, migration and invasion of thyroid cancer, such as BRAF, TERT, APC and CTNNB1 mutations; BRAF, ALK and NTRK fusion; as well as RET/PTC rearrangements. However, the most common type of genetic mutation is BRAF V600E. The solid tumor is highly differentiated, minimally malignant and often has a favorable prognosis; therefore, the best treatment for PTC is surgical resection with the 5-year survival rate up to 90%.^[16–18]^ It was reported that up to 90% of patients had their thyroid removed by surgery, which may lead to additional medical costs and psychological stress.^[19–21]^ The most common metastatic sites of thyroid cancer are lungs and bone with extremely rare metastases in brain^[22,23]^ This study reports the first case of solitary brain metastasis from occult papillary thyroid carcinoma in a Chinese Tibetan patient.

## 2 Case presentation

A 57-year-old Tibetan male patient came to the hospital with numbness in the right limb for 3 weeks. The examination revealed that the patient had normal development, adequate nutrition, no obvious acute or chronic medical conditions, and no obvious abnormalities in heart, lungs, liver, gallbladder, pancreas, spleen, and kidney. The bilateral Babinski signs were negative, and there were no other special manifestations, except for the right limb muscle strength (grade IV) and a protruding mass on the left forehead for 2 years. The CT, MRI, color ultrasound diagnosis, ultrasound-guided fine-needle aspiration and hematoxylin-eosin staining staining were used to for morphological examination. The specific expression of thyroid cancer-related proteins (cytokeratin, CK; thyroglobulin, Tg and thyroid transforming factor-1, TTF-1) were detected by the immunohistochemical analysis. The mutation of BRAF gene was found by amplification refractory mutation system-polymerase chain reaction, Sanger sequencing and next generation sequencing (NGS) analyses.

**Figure 1:**
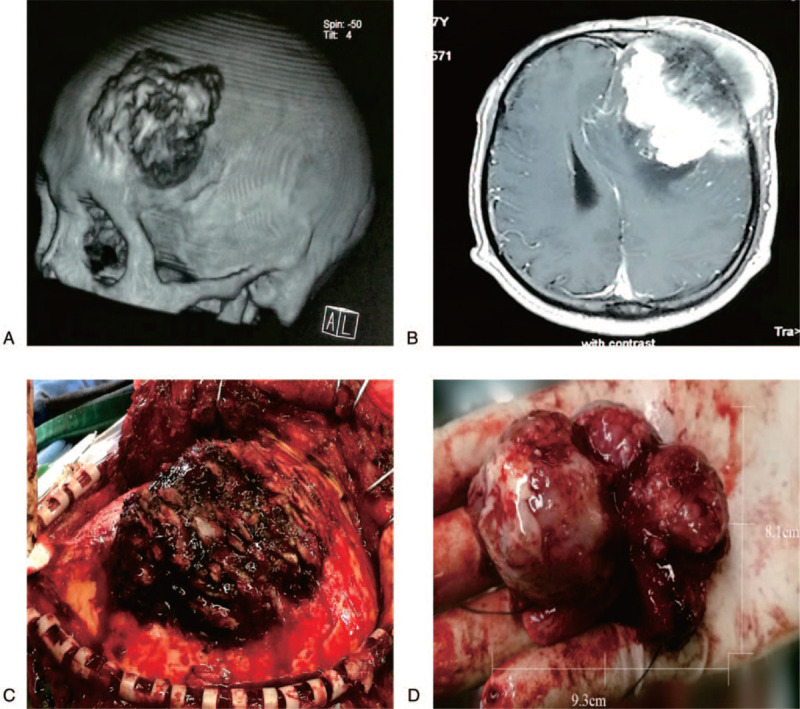
Preoperative skull CT/MRI scan and intraoperative photographs during resection and debulking. (A: Irregular bone destruction of the left frontal bone, the range is about 5.7 cm × 6.3 cm. B: Irregular masses are seen in the left frontal cranial and subscalp, the size is about 4.9 cm × 5.4 cm, it is uneven and obviously strengthened, and the center line structure is skewed to the right. C: The outer plate of skull, raised bone (4.8 cm × 5.2 cm) surface of the skull's outer plate is uneven, showing cancellous bone. D: The tumor isolated from the operation is hard in texture, about 5.7 cm × 6.3 cm in size.).

**Figure 2:**
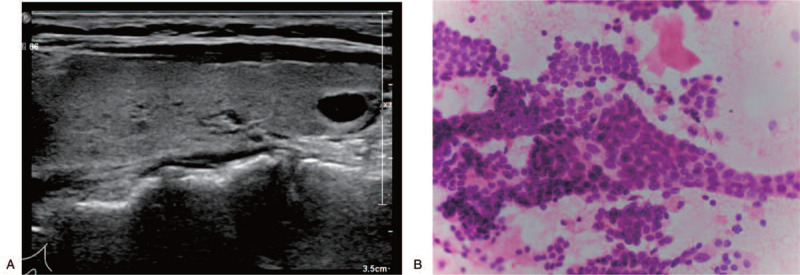
Preoperative conventional color ultrasound diagnosis (A) and ultrasound-guided fine-needle aspiration diagnosis (B). (A: The echo in the right lobe was uneven, and a 0.7 cm × 0.5 cm mass was visible. B: A biopsy specimen of the thyroid nodules was obtained (hematoxylin-eosin, 400×.).

The preoperative skull CT and MRI scan showed a 9.3 cm × 8.1 cm mass in the left frontal cortex (Fig. 1A and B). The skin on the surface of the mass was normal and hard in texture. The preoperative conventional color ultrasound diagnosis showed that the size of the bilateral thyroid was normal, and the echo in the right lobe was uneven. Left frontal lobe metastasis (thyroid cancer) resection with craniectomy and artificial skull repair” was suggested by the multi-disciplinary team (MDT). During the operation we observed that the scalp tissue and the left frontal skull were adhered, and the surface of the protruding bone tissue was uneven and loose. The hyperplasia of the inner and outer plates of the skull could be seen (Fig. 1C). The tumor tissue was white and solid, and there was no adhesion with the surrounding brain tissue (Fig. 1D). After the surgery, the completely excised tumor tissue was sent to the Clinical Molecular Medicine Testing Center for molecular pathology diagnosis. The preoperative conventional color ultrasound diagnosis showed that the size of the bilateral thyroid was normal, and the echo in the right lobe was uneven. A mass (0.7 cm × 0.5 cm) was visible, with oval shape and clearly defined border (Fig. 2A). Ultrasound-guided fine-needle aspiration was used to biopsy the thyroid nodules. The results showed that these tumor cells were relatively uniform in size with round nuclei, and small nucleoli was observed in some tumor cells. (Fig. 2B).

**Figure 3:**
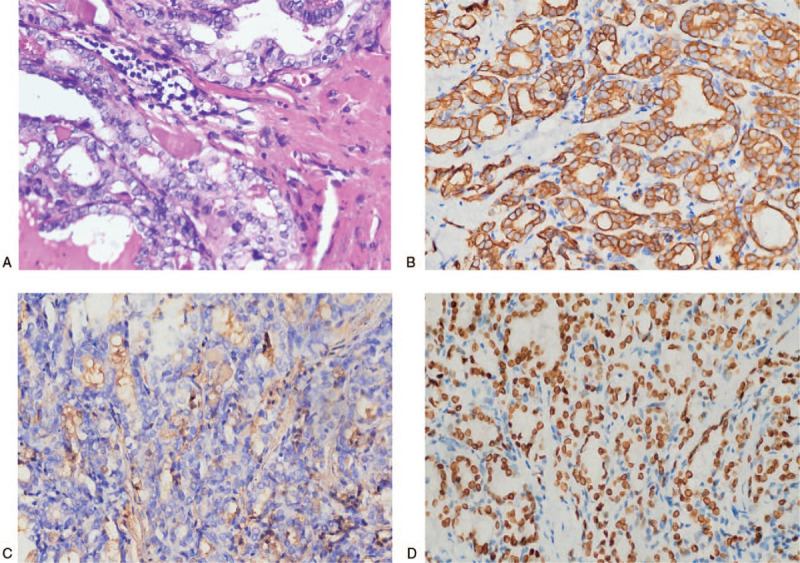
The hematoxylin-eosin staining and immunohistochemical (IHC) analysis staining of the brain metastases (400×). (A: Tumor cells are relatively uniform in size, with round nuclei, small nucleoli visible in part, cytoplasmic staining and mitotic divisions that are not easily seen. B: CK19 staining showed brownish yellow particles on the cell membrane (+++). C: TG staining showed brownish yellow particles on the cell membrane (+). D: TTF1 staining showed nucleus showing brownish yellow particles (+++).).

**Figure 4:**
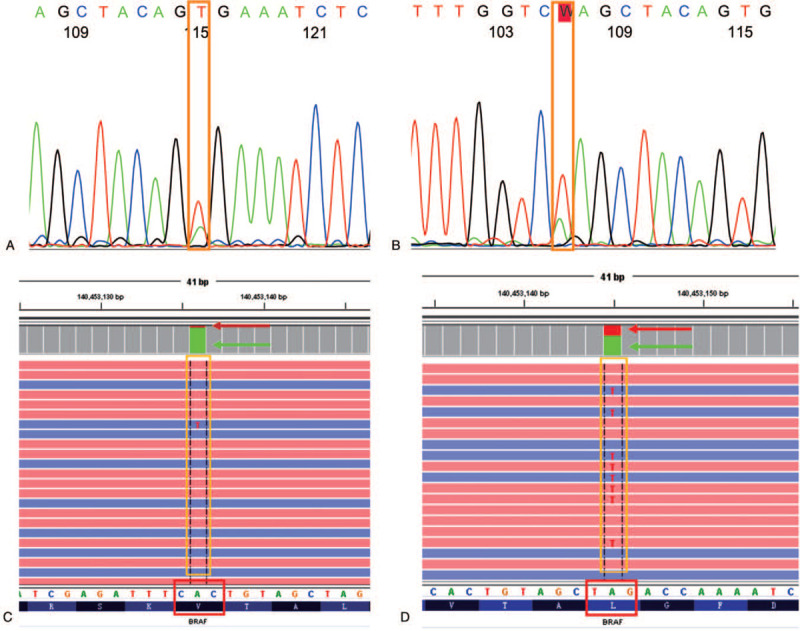
Genetic diagnosis results. (A: BRAF V600E (c.1799T>A) mutation tested by Sanger sequencing in situ. B: BRAF L597Q (c.1790T>A) mutation tested by Sanger sequencing in metastases. C: BRAF V600E (c.1799T>A) mutation tested by NGS in situ, and D: BRAF L597Q (c.1790T>A) mutation tested by NGS in metastases.).

Hematoxylin-eosin staining staining of the brain specimen showed that the nucleus of the cancer cells had the texture of the ground glass. The nuclear grooves and pseudoinclusions in the nucleus were clearly observed, and psammoma bodies were seen in the interstitial tissue (Fig. 3A). In general, the cytokeratin (CK), thyroglobulin (Tg) and thyroid transforming factor-1 (TTF-1) in papillary thyroid carcinoma were strongly immunohistochemically positive, in this patient. The immunohistochemical staining showed that the CK, Tg and TTF-1 were immunoreactive (Fig. 3B, C and D). The amplification refractory mutation system-polymerase chain reaction, Sanger and NGS analyses showed that the patient had double mutations of BRAF L597Q and V600E in two separate lesions (Fig. 4). The BRAF V600E (chr7:140453136 c.1799T>A) mutation was located in situ of thyroid cancer (Fig. 4A and C), but the BRAF L597Q (chr7:140453145 c.1790T>A) mutation was located in the brain metastases (Fig. 4B and D). The abundances of BRAF L597Q and V600E were 36.9% and 8.1% which was determined by NGS, respectively, and they were successfully verified by the gold standard for gene sequencing (Sanger sequencing).

In this rare case report, all the above findings supported the diagnosis of solitary brain metastasis of occult papillary thyroid carcinoma. There were no adverse and unanticipated events during the peri-operative period. During the 24-month follow-up, no postoperative complications or recurrence and metastasis were observed.

## 3 Discussion

According to the recommendations of the American Thyroid Association (ATA), FNAB is currently the most accurate method to identify benign or malignant thyroid nodules. However, there are still some cases that cannot be defined as benign or malignant,^[24]^ because the diameter of the thyroid nodules is less than 1 cm. Benign or malignant cells related to thyroid cancer can be detected by molecular markers, such as BRAF mutation, RAS mutation and RET/PTC rearrangement.^[25]^ Some guidelines (2017 Bethesda guidelines and 2019 NCCN) have recommended that molecular marker testing should be used for the assessment of benign or malignant thyroid nodules, especially in combination with genetic testing. The combined results could significantly improve the negative predictive value of cancer, which might reduce unnecessary surgery. One study reported that BRAF, PAX8/PPARγ and RET/PTC could increase the diagnostic specificity of PTC up to 100%.^[26]^ It was recognized that genetic testing was the prerequisite for individualized treatment and could be used to assess the sensitivity of targeted drugs, develop new drugs and explore combined drug treatment. Genetic testing could also help assess the risk of thyroid cancer recurrence and provide a basis for postoperative management. Comprehensive genomic analysis can identify new treatment paradigms to address the limited options and poor prognosis of patients with primary unknown site cancer. It is also possible to reduce the regular expenses and time-consuming search for the cancer's anatomic site of origin.^[27]^

However, genetic diagnosis cannot meet the clinical needs of including or excluding thyroid cancer and preoperative risk stratification of thyroid cancer. Although the positive results of FNA may be useful for individualized treatment of thyroid cancer, while the negative genetic results might not be an indicator of that cannot benefit from it.^[28]^

The BRAF V600E or other mutations (e.g., TERT promoter mutation or RET/PTC fusions and so on) could demonstrate mutations typical of classic PTC.^[29]^ In this study, 59 high-frequency mutations and targeted drug-related genes (e.g., BRAF, TERT, PTEN, TP53, PIK3CA, RAS, RET and so on) that may be related to thyroid cancer was determined by NGS, but the genetic test results showed that only BRAF gene mutations (L597Q and V600E in 2 separate lesions) occurred in the patient. Although the BRAF V600E (c.1799T>A) mutation is the most common type of genetic mutation in thyroid cancer, the BRAF L597Q (c.T1790A) mutation is extremely rare.

## 4 Conclusion

The rare BRAF L597Q (c.T1790A) point mutation, which is regarded as an oncogene, has been previously reported and described in childhood acute lymphoblastic leukemia.^[30,31]^ Additionally, other studies have reported that the rare BRAF (c.T1790A) point mutation might have some correlation with tumor metastasis in melanomas and lymphoblastic leukemia.^[32,33]^ Therefore, we hypothesize that the BRAF L597Q mutation might play a specific role in inducing the solitary brain metastasis of occult papillary thyroid carcinoma in Chinese Tibetan patient, and a large number of functional experiments and clinical studies is needed to confirm the decisive role of BRAF L597Q mutation in the brain metastasis of occult thyroid cancer. In addition, we hope our research will lead to the development of targeted therapeutics in future.

## Author contributions

YW and XSL designed the study and take responsibility for the integrity and accuracy of the data analysis. LC, HLB and HDL performed the main experiments and analysed the data; XSL drafted and revised the manuscript. All authors contributed to this manuscript. All authors read and approved the final manuscript.

**Conceptualization:** Xiaosong Li.

**Methodology:** Huili Bai, Huandong Liu.

**Resources:** Huandong Liu.

**Software:** Yue Wu.

**Writing – original draft:** Ling Chen, Yue Wu.

**Writing – review & editing:** Xiaosong Li.

## Abbreviations

Abbreviations: NGS = next generation sequencing, PTC = papillary thyroid carcinoma, US-FNA = ultrasound-guided fine-needle aspiration.
